# Breaking the Silence: Willingness to Intervene in Cases of Intimate Partner Violence

**DOI:** 10.3390/bs15121680

**Published:** 2025-12-04

**Authors:** Nika Hober, Vanja Ida Erčulj

**Affiliations:** Faculty of Criminal Justice and Security, University of Maribor, 1000 Ljubljana, Slovenia; nikaa.hober@gmail.com

**Keywords:** IPV, women, victims, attitudes towards IPV, willingness to report

## Abstract

Intimate partner violence (IPV) is frequently regarded as a private issue, with witnesses often responding accordingly. As a result, a lot of violence remains unreported, either by the victim or observers. Statistical analyses were conducted on data from an online survey of 600 respondents aged 18–65 in Slovenia. This research aimed to examine the influence of demographic factors and attitudes toward IPV on willingness to intervene or report IPV to the police. A theoretical model of intervention in IPV cases was developed and tested using structural equation modeling. The role of attitudes towards IPV as mediators between demographic variables and willingness to intervene in cases of IPV was investigated by comparing the difference in model fit between models that presumed partial and full mediation. The results supported a full mediating role of attitudes towards IPV. A full mediation model fitted the data well (SB χ^2^ = 765.8; df = 320; *p* < 0.001; SB χ^2^/df = 2.4; RMSEA = 0.048 (90% CI: 0.044–0.053); NFI = 0.93; NNFI = 0.95; CFI = 0.96; IFI = 0.96; SRMR = 0.08). With increasing age, and for men, the tolerance of psychological IPV increases. Men and those with lower education are more tolerant of physical IPV. Individuals with higher tolerance for physical or psychological IPV were less likely to intervene or contact the police, and more likely to remain passive. Perceptions of police effectiveness were not related to willingness to intervene. The findings emphasize the pivotal role of attitudes towards IPV, underscoring the importance of raising awareness of the phenomenon and encouraging individuals to “break the silence” by reporting it to the police.

## 1. Introduction

Intimate partner violence (IPV) is any behavior in intimate relationships—marital, extramarital, or dating—that causes physical, psychological, or sexual harm to a partner. This includes physical, sexual, and psychological aggression, abuse, or any controlling behavior (Patra et al., 2018; WHO, 2024). Most IPV and sexual violence cases involve male perpetrators and female victims. One in three women has experienced physical and/or sexual violence by a partner or by others at least once (WHO, 2021).

The 2020 pandemic again highlighted the urgent need to treat violence against women as a public health issue (WHO, 2021). COVID-19 control measures, such as lockdowns and social distancing, led to more reports of domestic violence, especially IPV against women, which rose worldwide during the pandemic (UN WOMEN, 2020; WHO, 2021). IPV usually persists for years, not as isolated events (FRA, 2014).

The prevalence of IPV varies across different studies (Alhusen et al., 2023). Violence against women is a widespread human rights violation across the European Union. About 22% of women in relationships have experienced physical or sexual violence, with roughly one-third of these reporting repeated rape. Psychological abuse is also common, affecting 43% of women by a current or former partner, and one in six victims continues to experience violence even after the relationship ends (FRA, 2014). The National Intimate Partner and Sexual Violence Survey, a continuous nationally representative study of U.S. adults, provides data on lifetime and past-year prevalence of various forms of IPV among women and men. The latest findings show that nearly half of U.S. women (59 million) have experienced lifetime IPV, while 7.3% (9 million) were victims in the past year (Leemis et al., 2022). The first global systematic review of scientific data on the prevalence of two forms of violence against women was published in 2013. Based on data from around the world, it presents for the first time global and regional estimates of the prevalence of both IPV and non-partner sexual violence. 35% of women worldwide have experienced physical and/or sexual violence, mostly from their partner. Among women in relationships, almost a third experience IPV, and in some regions the figure is as high as 38%. Intimate partners commit nearly 40% of female homicides globally (WHO, 2013). Global estimates of the prevalence of IPV for 2018 (based on data from 2000 to 2018) indicate that it is considerable over a woman’s lifetime. 26–27% of women aged fifteen or older who have ever been married or in a relationship have experienced physical and/or sexual violence by a current or former partner, which means between 641 and 753 million women worldwide. These estimates also suggest that IPV begins early, even among young girls. Regional estimates for 2018 show that lifetime prevalence of physical and/or sexual IPV among women aged 15 to 49 who have ever been partnered is highest in the least developed countries (three subregions of Oceania). Europe, Central, Eastern, and South-Eastern Asia, as well as Australia and New Zealand, have the lowest rates. In the last 12 months, the prevalence of IPV among ever-married women has been highest in the least developed countries and subregions of Oceania (WHO, 2021).

IPV against women is a hidden phenomenon characterized by low reporting rates, and there are limited studies examining the willingness to report IPV to authorities (Leon et al., 2021). The level of tolerance and acceptance of IPV is reflected in the willingness to intervene (Gracia et al., 2018). It is often said that IPV occurs behind closed doors, but research shows that bystanders are sometimes present. Bystanders may respond in different ways—they may do nothing, support the victim, or side with the perpetrator (E. Taylor et al., 2016). Research also shows that most victims speak about violence to at least one informal support person (such as family or friends). This is associated with various demographic, interpersonal, and situational factors (Sylaska & Edwards, 2014). Literature further suggests that bystander reactions generally fall into two categories: supportive or mediating actions, such as offering help or advice, and formal actions, such as reporting to authorities. Silence or inaction is also a possible response to incidents of IPV against women (Gracia et al., 2018; Sylaska & Edwards, 2014). Even when aware of IPV, individuals often do not intervene (Gracia & Herrero, 2006).

Attitudes play a central role in understanding the behavior of a bystander of IPV. They may pertain to specific actions or to the targets of those actions, such as reporting partner violence or perspectives on the issue (Gracia & Herrero, 2006). Individuals from countries with higher perceived prevalence of IPV against women and higher gender equality prefer formal responses. In comparison, countries with lower perceived prevalence and negative perceptions of the appropriate response prefer informal responses (Gracia & Herrero, 2006; Serrano-Montilla et al., 2020). People are more willing to intervene and report violence when the abuse appears to be more severe (Ermer et al., 2021; Leon et al., 2021). In cases of physical and sexual violence, it is expected that the victim should respond by reporting the incident to the police (C. A. Taylor & Sorenson, 2005). Also in Slovenia, the country in which the current study was conducted, the previous research shows that a victim of IPV tends to report violence to the police after severe and long-term abuse (Prislan et al., 2022). A victimization study, conducted by the Statistical Office of the Republic of Slovenia, reveals that most people did not report IPV to the police (Statistical Office of the Republic of Slovenia, 2020). Victims mainly found confidants in their family members, relatives, and friends (56%), while the proportion who reported IPV to police or talking to medical staff was about 21%. An extensive meta-analytic study (Stith et al., 2004) identified five key factors associated with physical IPV, namely emotional abuse (psychological violence), forced sex, illicit drug use, attitudes regarding marital violence, and marital satisfaction. Attitudes towards IPV are therefore one of the key factors in the manifestation of violence in intimate partner relationships. Alfredsson et al. (2014) further conclude that the strength of personal norms and emotions when facing IPV are predictive factors for intervention. A sense of personal responsibility significantly shapes public responses to IPV. Police reporting is generally viewed as appropriate only in cases of severe, repeated, or extreme violence, while less severe cases are seen as better addressed through mediation or tolerance (Gracia et al., 2009). Willingness to intervene is closely linked to attitudes toward violence. Men perceive IPV as less severe (Willis-Esqueda & Delgado, 2020). Attitudes found to be closely related to higher tolerance of IPV are sexist beliefs, victim-blaming, justification of the perpetrator, acceptance of rape myths, and endorsement of traditional gender roles (Badenes-Sastre et al., 2024). Sexist attitudes toward women are associated with negative intentions to help victims (Cinquegrana et al., 2017; Pacilli et al., 2016). Inconsistent results are found regarding gender and willingness to report violence (Leon et al., 2021).

The higher frequency and lower tolerance of IPV are positively associated with willingness to intervene (Gracia & Herrero, 2006; Serrano-Montilla et al., 2020). Lower interference was related to victim-blaming attitudes—when victims are seen as responsible for their victimization, the likelihood of others offering help decreases (Pavlou & Knowles, 2001). Several studies have identified a link between willingness to intervene and the victim’s infidelity (Cinquegrana et al., 2017; Pagliaro et al., 2020; Witte & Mulla, 2012). When a victim is perceived as being the culprit or triggering a violent response from their partner (such as in the case of infidelity), people are less willing to intervene. Such perceptions are closely linked to perceptions of traditional male sexual roles in society.

Prior research shows that attitudes toward IPV are shaped by socio-demographic factors such as age, education, gender, and income. Later-life cohorts have been found to adopt more traditional and tolerant views, perceiving violence as more acceptable, though some studies report that young adults can also be more tolerant. In contrast, others identify lower tolerance among those aged 26–35 (Gracia et al., 2009; Prado Rivera et al., 2024; Tran et al., 2016; Worden & Carlson, 2005). Previous research suggests that women tend to exhibit lower tolerance toward violence and a greater willingness to intervene compared to men (Alzoubi & Ali, 2021; Gracia et al., 2020; Prado Rivera et al., 2024). However, contrasting findings indicate that women—particularly those who have experienced intimate partner violence—may demonstrate higher levels of tolerance for violent behavior (Copp et al., 2019; Khawaja et al., 2008). Education and income are consistently associated with less tolerance, as higher levels of both predict greater awareness of violence and rejection of violence-supportive attitudes (Badenes-Sastre et al., 2024; Copp et al., 2019; Reyal et al., 2020). Moreover, individuals from more privileged backgrounds tend to show lower tolerance of violence (Copp et al., 2019). Despite these findings, evidence remains mixed, highlighting the need for further investigation into how socio-demographic determinants shape attitudes toward IPV.

**Figure 1:**
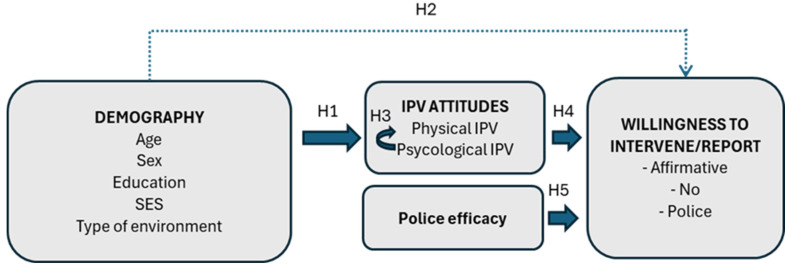
The theoretical model of intervention in the case of intimate partner violence (IPV).

This study aims to further explore the role of demographic factors and attitudes towards IPV in the willingness to intervene and report cases of IPV to the police. Furthermore, the role of perceived police efficacy in cases of dealing with IPV and willingness to intervene will be examined. The link between the perception of police effectiveness in cases of IPV and the willingness to report such violence to the police has not yet been studied, to our knowledge. The theoretical model of willingness to intervene explored in our study is presented in Figure 1.

The following hypotheses will be tested:

**H1:**  *There is a relationship between demographic variables and attitudes towards IPV.*

**H1a:**  *There is a relationship between age and attitudes towards IPV.*

**H1b:**  *There is a relationship between sex and attitudes towards IPV.*

**H1c:**  *There is a relationship between educational level and attitudes towards IPV.*

**H1d:**  *There is a relationship between SES and attitudes towards IPV.*

**H1e:**  *There is a relationship between the type of environment and attitudes towards IPV.*

**H2:**  *There is a relationship between demographic variables and willingness to intervene.*

**H3:**  *There is a relationship between attitudes towards psychological violence and attitudes towards physical IPV.*

**H4:**  *There is a relationship between attitudes towards IPV and willingness to intervene.*

**H5:**  *There is a relationship between perceived police effectiveness in IPV and willingness to intervene.*

Willingness to intervene is a particularly relevant topic, as it remains relatively unexplored and warrants further scholarly attention. It is unclear whether full or partial mediation (through attitudes towards IPV) exists between demographic variables and willingness to intervene.

## 2. Materials and Methods

### 2.1. Participants

The final sample consisted of 600 adults aged 18 to 65 years in Slovenia. Half of them (*n* = 300) were women, and nearly half (*n* = 296; 49%) had a higher education level (at the university level). Mean age (*SD*) was 44.4 (13.4) years. More than half (337; 56%) lived in an urban environment, and according to their household or personal income, 177 (29.5%) had a lower socioeconomic status (SES).

### 2.2. Procedure

The data in this paper were collected as part of a broader study on attitudes toward intimate partner violence, willingness to intervene in cases of violence, and attitudes toward minority groups. The data collection was conducted in August 2023, involving 613 participants aged 18 to 65. The final sample of the current study comprised 600 participants who provided complete answers to all the investigated variables in the structural equation modeling. The sampling frame consisted of members from the online panel JazVem (Valicon, 2024), which comprises over 20,000 members from diverse demographic groups. A combination of stratified random and quota sampling was employed, and the data were weighted to ensure that the demographic structure of the sample reflected that of the general population. Strata were defined according to gender, age, and education. The response rate was 27%.

The study was approved by the Ethics Committee of the Faculty of Criminal Justice and Security (approval number 1909-2023).

### 2.3. Questionnaire Development and Measures

The intervention scale was adopted from Willis-Esqueda and Delgado (2020). All items were measured on a five-point Likert scale of agreement. Higher values indicated higher agreement with the statement. The scale consisted of four dimensions:

▪ Affirmative intervention: This dimension refers to the expectation that people close to the victim, or even bystanders, should intervene in cases of IPV. Initially, it was measured by six items: “A neighbor should intervene if he or she witnesses domestic violence,” “A friend should intervene if he or she witnesses domestic violence,” “I would intervene if I witnessed domestic violence,” “A family member should intervene if he or she witnesses domestic violence,” “A passerby should intervene if he or she witnesses domestic violence,” and “A neighbor/friend should intervene if he or she sees signs of child abuse.” In our study, only the first five items loaded substantially on a single factor and were therefore retained for the analysis (Cronbach’s α = 0.91).
▪ Police intervention: This type of intervention underscores the necessity of law enforcement involvement in IPV situations. It was measured by four items (Cronbach’s α = 0.88): “An individual should contact the police if they are threatened with a weapon,” “An individual should contact the police after his or her partner is physically abusive,” “An individual should contact the police if abuse occurs when a child is present,” and “An individual should contact the police if their partner threatens abuse.”
▪ No Intervention: This dimension captures the belief that IPV is a private matter and that outside parties should only intervene in extreme circumstances or with clear evidence. The initial scale comprised seven items, with one example being “An individual shouldn’t contact the police unless they have proof of a domestic assault.” Following factor validation, only two items were retained for analysis due to poor loadings of others (Cronbach’s α = 0.98): “Verbal arguments with threats of physical harm by the man *to his partner* should not be reported to the police,” and “Verbal arguments with threats of physical harm by the woman *to her partner* should not be reported to the police.” Modifications were made to these statements to clarify their reference to IPV (the words in italic text were added).
▪ Intervention threshold: his aspect was measured initially with three items: “An individual should contact the police if their partner threatens abuse,” “A man should be automatically arrested for any signs of abuse on a woman,” and “A woman should be automatically arrested for any signs of abuse on a man.” However, only the latter two items exhibited significant loadings on a unified factor. Since this content did not reflect a willingness to intervene but relatively strong views regarding formal consequences of IPV, we excluded this scale from our analysis.

The Attitude towards Violence Scale assessed perspectives on both physical and psychological IPV, with responses provided on a five-point Likert scale of agreement, where higher values signified greater tolerance for IPV. The scale was divided into two dimensions:

▪ Attitudes towards physical violence: Based on research by Davidson and Canivez (2012), this included statements like, “It is all right for a partner to hit the other if they are unfaithful,” “It is all right for a partner to slap the other if insulted or ridiculed,” “It is all right for a partner to slap the other’s face if challenged,” and “It is all right for a partner to hit the other if they flirt with others” (Cronbach’s α = 0.91).
▪ Attitudes towards psychological IPV: Developed from the WHO questionnaire on IPV, this dimension included statements such as, “It is acceptable for a partner to constantly insult the other privately,” “constantly humiliate the other in front of others,” “threaten to hurt the other partner,” and “constantly belittle the other partner” (Cronbach’s α = 0.91).

The measure of Police Efficacy in domestic violence cases was created by the authors, consisting of four items (Cronbach’s α = 0.83): “Police intervention in domestic violence cases is swift (officers arrive at the scene quickly),” “Police intervention in domestic violence cases is effective (the violence is successfully stopped),” “The police can successfully intervene in cases of domestic violence,” and “Police interventions effectively stop domestic violence.”

To ensure clarity and accuracy, the scales adopted from other authors were localized through a translation and back-translation process. Two independent translators first translated the questionnaire into Slovene, and after reaching a consensus, a third translator translated it back to the original language. The meanings of items post-back-translation were compared with the original items to ensure consistency.

### 2.4. Statistical Analysis

Exploratory and confirmatory factor analysis were performed in the first step to validate the measurement model, and in the second step, the structural equation model was constructed. By following this approach, we adhered to the two-step method suggested by Anderson and Gerbing (1988). A robust maximum likelihood method of parameter estimation (Boomsma & Hoogland, 2001) was used to evaluate the measurement and structural models.

The sign of convergent validity was statistically significant, with considerable (>0.50) loadings of items on the factor they were intended to measure (Anderson & Gerbing, 1988; Hair et al., 1998), and a good overall fit of the model (Steenkamp & van Trijp, 1991). Average variance extracted (AVE) above 0.50 was considered a sign of good construct validity.

Reliability was assessed by Cronbach’s alpha and composite reliability. For both, values above 0.60 were considered an indicator of sufficient reliability (Fornell & Larcker, 1981; Ursachi et al., 2015).

The model’s fit was evaluated using the Sattora-Bentler scaled Chi-Square, the chi-square to degrees of freedom ratio, Comparative Fit Index (CFI), Incremental Fit Index (IFI), Non-Normed Fit Index (NNFI), the Root Mean Square Error of Approximation (RMSEA), and the Standardized Root Mean Square Residual (SRMR). Values of 0.95 and above, or 0.90 and above for CFI, NNFI, and IFI, and values of 0.08 and below for RMSEA and SRMR, indicate a good model fit (Hu & Bentler, 1998). The chi-square to degrees of freedom ratio between 1 and 3 indicates a good overall fit of the model (Baumgartner & Homburg, 1996).

Partial mediation was tested by comparing the model with only indirect paths between the demographic variable and the dependent variable with the model including both direct and indirect paths. Sattora-Bentler (SB) scaled chi-square difference test was used to compare the two models (Satorra, 2000).

LISREL 12.0 (Scientific Software International, Inc., Chapel Hill, NC, USA) was used for model calibration and hypothesis testing. SPSS, v. 29 (IBM Corp., New York, NY, USA), was used to calculate Cronbach’s alpha and descriptive statistics.

## 3. Results

Confirmatory factor analysis resulted in a good overall fit of the measurement model (SB χ^2^ = 529.9; df = 215; *p* < 0.001; SB χ^2^/df = 2.5, RMSEA = 0.049; NFI = 0.95; NNFI = 0.96; CFI = 0.97; IFI = 0.97; SRMR = 0.048). Standardized loadings, AVE, and composite reliability measures for multi-item factors are summarized in Table 1. Factor loadings are above 0.50 for all items. All loadings are statistically significant. The good overall fit of the model supports construct validity, which is further supported by an AVE above 0.50 for all the constructs. Composite reliability for all constructs is above 0.8, indicating good measurement reliability.

Descriptive statistics of constructs calculated as composite variables are presented in Table 2. The tolerance of IPV, in general, is low, and the willingness to intervene or report abuse to the police is high. The efficacy of police in cases of IPV is, on average, close to the midpoint on the evaluated scale.

In the structural equation model, the affirmative, police, and no intervention were dependent variables, demographic variables were independent variables, and attitudes towards physical and psychological violence were mediating variables. First, we compared the fit of the partial mediation model, which assumes both direct and indirect (through attitudes) relationships between demographic variables and the three intervention variables, to the model assuming only indirect relationships between demographic variables and the three intervention variables. The two models did not significantly differ in model fit (∆ SB χ^2^ = 6.6; df = 15, *p* = 0.967). As partial mediation did not significantly improve the model fit, the second hypothesis, which assumes a direct effect of demographic variables on willingness to report or intervene in cases of IPV, is rejected. The following presents only the results of the full mediation model.

**Figure 2:**
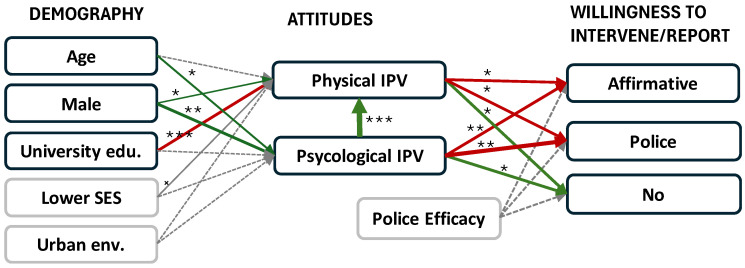
Structural equation model (green arrows = positive significant relationship; red arrows = negative significant relationship; gray dotted arrow = non-significant relationship; * *p* < 0.05; ** *p* < 0.01; *** *p* < 0.001; ^×^ *p* < 0.10; stronger relationship between variables is denoted with thicker arrows between constructs).

Structural equation model exhibited good overall fit (SB χ^2^ = 765.8; df = 320; *p* < 0.001; SB χ^2^/df = 2.4; RMSEA = 0.048 (90% confidence interval (CI): 0.044–0.053); NFI = 0.93; NNFI = 0.95; CFI = 0.96; IFI = 0.96; SRMR = 0.08). The simplified structural equation model is presented in Figure 2. The results support the first hypothesis, which posits a statistically significant relationship between certain demographic variables and attitudes towards IPV. With increasing age, and for men, the tolerance of psychological IPV increases (H1a and H1b). Men and those with lower education (H1c) are more tolerant of physical IPV. Socio-economic status is marginally statistically significantly related to attitudes towards physical violence (H1d), with people with lower SES showing higher tolerance of such violence. SES is not statistically significantly associated with psychological violence. The type of environment does not correlate with attitudes towards IPV (H1e). The results support the third hypothesis, as a statistically significant relationship was found between attitudes towards psychological and physical IPV. Demographic variables and attitudes towards psychological violence explain 57% of the variance in attitudes towards physical violence. The share of explained variance of attitudes towards psychological violence remains low (3%).

The results regarding the relationship between attitudes towards physical and psychological IPV and willingness to intervene or report the behavior to the police are very consistent for all three intervention factors—affirmative intervention, police intervention, and no intervention. Those with higher tolerance towards physical or psychological IPV are less inclined to intervene (affirmative intervention) or contact the police (police intervention) and more inclined not to intervene in case of IPV. The data therefore supports the fourth hypothesis. Perception of police efficacy is not connected to willingness to intervene or contact the police—the fifth hypothesis is not accepted. The attitudes towards violence explain 9% of the variance of affirmative intervention, 18% of the variance of police intervention, and 12% of the variance in no intervention.

## 4. Discussion

The main aim of the present study was to explore the relationship between demographic variables, attitudes towards IPV, and willingness to intervene in cases of IPV—either by intervening informally or reporting the IPV to the police. The literature review indicates inconclusive evidence regarding the role of demographic factors in willingness to intervene and attitudes towards IPV. Consistent findings pertain to gender differences in the tolerance of IPV, with men exhibiting higher tolerance of such behavior (Alzoubi & Ali, 2021; Gracia et al., 2020; Prado Rivera et al., 2024). Our study also confirmed this finding: men showed higher tolerance for physical and psychological IPV. The link between gender and IPV attitudes was stronger for psychological than for physical IPV. Nevertheless, mean values for attitudes towards psychological and physical violence were low (1.41 (95% CI: 1.36–1.46) and 1.64 (95% CI: 1.58–1.70), respectively), suggesting generally low tolerance in Slovenian society. Interestingly, respondents were more tolerant of physical than psychological violence. This can be explained by examining the content of the statements measuring attitudes towards physical and psychological violence. In the first case, the described violence is circumstantial, about situations where a victim might be seen as responsible for their partner’s violent reaction (e.g., infidelity, flirting). In the second case, long-term psychological abuse is described. Previous research has shown that greater blame is attributed to victims when they are perceived as triggering their partner’s violent reaction through their actions, particularly in cases of flirting or infidelity (Badenes-Sastre et al., 2024; Pavlou & Knowles, 2001). On the other hand, the frequency of IPV plays a crucial role in tolerance of violence as well as in willingness to intervene (Gracia & Herrero, 2006; Serrano-Montilla et al., 2020). This finding was also reflected in our study: tolerance of psychological IPV, when it is long-lasting and frequent, is lower than tolerance of violence (even physical) perceived as a one-time, circumstantial event. Research in Slovenia from 2005 (Medarić, 2011) likewise showed higher tolerance of one-time or rare violent events in intimate partner relationships (e.g., slapping or hitting a partner when jealousy is present).

Our findings also align with previous evidence that tolerance of IPV is associated with a lower willingness to intervene. Individuals showing higher tolerance towards physical or psychological violence were less willing to intervene (affirmative intervention), less supportive of police intervention in cases of domestic violence (formal intervention), and more inclined to ignore the situation by perceiving IPV as a private matter (no intervention). For police intervention—a formal response to IPV—a stronger relationship was found with attitudes towards psychological IPV than with attitudes towards physical IPV. This finding is supported by previous studies showing higher willingness to intervene in cases of long-term and frequent abuse (Gracia & Herrero, 2006; Prislan et al., 2022; Serrano-Montilla et al., 2020), which in our study was more closely reflected in indicators measuring psychological than physical violence.

The data further indicate a clear preference for formal over informal intervention (4.6; 95% CI: 4.56–4.64 and 4.26; 95% CI: 4.21–4.31, respectively). This pattern is consistent with evidence from countries with higher gender equality and perceived IPV prevalence (Gracia & Herrero, 2006; Serrano-Montilla et al., 2020). In Slovenia, the perceived prevalence is slightly above 80% (Vázquez et al., 2021), while the country ranks 12th in the EU in the Gender Equality Index as of 2024 (European Institute for Gender Equality, 2025).

Despite these findings, only about 20% of the variance in willingness to intervene was explained by attitudes, leaving a significant portion unexplained. Demographic factors did not exert a direct effect but operated through attitudes. Contrary to expectations, perceptions of police effectiveness were not predictive, unlike in Chile, where awareness of the degree to which the law legally prosecutes violent behaviors was a key predictor of choosing the police as a source of help (Juarros-Basterretxea et al., 2024).

Demographics nonetheless shaped IPV attitudes: men, older adults, and less-educated individuals were more tolerant of violence. Age and education emerged as significant predictors, consistent with studies linking traditional values and lower education to greater tolerance of IPV (Copp et al., 2019; Prado Rivera et al., 2024; Tran et al., 2016; Worden & Carlson, 2005). SES showed only marginal effects. More importantly, tolerance of psychological violence strongly predicted tolerance of physical violence, consistent with evidence identifying emotional abuse as a risk factor for physical IPV (Stith et al., 2004).

### Limitations and Future Directions

Several limitations should be considered when interpreting the findings. Respondents were not provided with a formal definition of IPV/domestic violence at the outset of the survey. We relied on participants’ existing understanding of the term, which may vary and may be more strongly anchored in physical forms of violence than in psychological abuse (Sivarajasingam et al., 2022). This could have influenced responses, particularly on items framed in general terms. Future studies should provide a brief standardized definition and/or use clearly differentiated scenario-based items for psychological and physical IPV.

Online surveying was the most appropriate data collection method for sensitive topics such as the one under study. However, online panels have certain limitations (Blom et al., 2017), primarily related to the exclusion of individuals with lower levels of digital literacy and to self-selection—individuals willing to participate in surveys may differ systematically from those who are not, even when probability sampling within strata is applied. Nevertheless, self-selection and nonresponse can also be problematic in probability samples. Given the sensitivity of the studied topic and to reduce the risk of socially desirable responses, online surveying was still the most suitable data collection method in our case.

The cooperation rate (27%) falls within common ranges for panel surveys (Kennedy et al., 2016; Mercer et al., 2017; Yeager et al., 2011), but remains relatively low, and nonresponse bias cannot be fully ruled out. Therefore, generalization to the broader Slovenian population should be made with caution.

The Police Intervention Scale, adopted from Willis-Esqueda and Delgado (2020), includes two items describing aggravating circumstances not specifically linked to IPV (threat with a weapon; presence of a child). Although all items loaded on a single factor, these scenarios may capture endorsement of formal intervention under perceived high severity rather than IPV-specific reporting alone, potentially inflating support for police involvement. Future work should separate “core IPV” scenarios from severity-escalation scenarios to test whether they represent distinct reporting thresholds.

As a large portion of the variance in attitudes towards psychological violence remains unexplained, further research in this area is needed. Other study findings suggest that IPV is mainly perceived as physical violence between partners, typically with the woman being a victim (Sivarajasingam et al., 2022). Additionally, in Slovenia, qualitative research reveals the normalization of IPV in rural areas and difficulties in recognizing other types of IPV, such as non-physical IPV, among healthcare and social services professionals (Knežević Hočevar, 2016). When investigating psychological IPV, broader cultural factors—such as sexist beliefs, social norms, and traditional views of gender roles—should be included in future models (Gracia et al., 2020). Additional risk factors, such as substance abuse, childhood exposure to violence, and marital satisfaction, also warrant attention (Stith et al., 2004).

As a large portion of the variance in willingness to intervene remained unexplained in our study, future research should consider additional factors, such as knowledge of legislation and prior contact with protection systems, which have been identified as essential predictors elsewhere (Kim & Ferraresso, 2022; Youstin & Siddique, 2019).

Finally, because the design is cross-sectional and based on self-report, causal inference is limited. While the mediation model is theoretically grounded, longitudinal or experimental designs are needed to confirm the temporal ordering of demographic factors, attitudes, and intervention behavior.

## 5. Conclusions

This study provides a population-based examination of attitudes toward psychological and physical IPV and its links to intervention orientations in Slovenia. The SEM results indicate an indirect pathway in which socio-demographic characteristics relate to willingness to intervene largely through their association with IPV attitudes.

Consistent with hypotheses derived from the prior literature, men and older respondents expressed higher tolerance of both psychological and physical IPV. Lower education was also associated with higher tolerance, while SES showed weaker and less consistent effects. Tolerance of psychological IPV strongly predicted tolerance of physical IPV, highlighting the central role of non-physical abuse in shaping wider normative acceptance of partner violence.

Across all intervention outcomes, attitudes toward IPV were the most stable predictors: higher tolerance was associated with lower endorsement of informal intervention and police reporting, as well as stronger agreement that IPV should remain a private matter. Although overall tolerance was low and willingness to intervene high, IPV remains substantially underreported, including in Slovenia (Filipčič et al., 2021). This underscores the need for sustained public education, particularly on psychological IPV, and for clear, accessible reporting pathways.

Given methodological constraints, these findings should be interpreted as a context-specific baseline rather than a definitive causal account. Even so, they suggest that prevention efforts may be most effective when targeting normative acceptance of psychological IPV, since these beliefs are closely tied to both tolerance of physical violence and elevated intervention thresholds. Future research should utilize refined scenario measures, incorporate a safe assessment of IPV exposure, and examine the cultural and situational drivers of bystander action and help-seeking.

## Figures and Tables

**Table 1:** Standardized loadings, AVE, and composite reliability (CR) for multi-item constructs (results of confirmatory factor analysis).

	Std. Loading
Attitudes towards psychological violence (CR = 0.94; AVE = 0.80)	
It is all right for the partner to…	
constantly insult the other privately	0.85
constantly humiliate the other in front of others	0.93
threaten to hurt the other partner	0.91
constantly belittle the other partner.	0.88
Attitudes towards physical violence (CR = 0.91; AVE = 0.73)	
It is all right for the partner to…	
hit the other if they are unfaithful.	0.82
slap the other if insulted or ridiculed.	0.90
slap the other’s face if challenged.	0.88
hit the other if they flirt with others.	0.81
Affirmative intervention (CR = 0.91; AVE = 0.68)	
A neighbor should intervene if he or she witnesses domestic violence.	0.8
A friend should intervene if he or she witnesses domestic violence.	0.7
I would intervene if I witnessed domestic violence.	0.78
A family member should intervene if he or she witnesses domestic violence.	0.91
A passerby should intervene if he or she witnesses domestic violence.	0.91
Police intervention (CR = 0.89; AVE = 0.68)	
An individual should contact the police if he or she is threatened with a weapon.	0.79
An individual should contact the police after his or her partner is physically abusive.	0.92
An individual should contact the police if abuse occurs when a child is present.	0.85
An individual should contact the police if his or her partner threatens abuse.	0.72
No Intervention (CR = 0.92; AVE = 0.85)	
Verbal arguments with threats of physical harm by the man to his intimate partner should not be reported to the police.	0.9
Verbal arguments with threats of physical harm by the woman to her intimate partner should not be reported to the police.	0.94
Police effectiveness (CR = 0.83; AVE = 0.55)	
Police intervention in cases of domestic violence is swift (officers arrive at the scene quickly).	0.69
Police intervention in cases of domestic violence is effective (the violence is successfully stopped).	0.88
The police can successfully intervene in cases of domestic violence.	0.63
Police interventions successfully stop domestic violence.	0.75

**Table 2:** Descriptive statistics of constructs.

	*n*	Min	Max	Mean	SD
Attitudes towards physical violence	600	1	5	1.64	0.73
Attitudes towards psychological violence	600	1	5	1.41	0.6
Affirmative intervention	600	1	5	4.26	0.64
Police intervention	600	1	5	4.6	0.53
No intervention	600	1	5	1.97	0.87
Police Efficacy	600	1	5	2.88	0.81

## Data Availability

The data set is available on Zenodo: https://doi.org/10.5281/zenodo.17413944, accessed on 22 October 2025.

## Abbreviations

The following abbreviations are used in this manuscript:

IPV	Intimate partner violence
SES	Socio-economic status
RMSEA	Root mean square error of approximation
CFI	Comparative fit index
WHO	World Health Organization
FRA	European Union Agency for Human Rights
NFI	Normed Fit Index
NNFI	Tucker–Lewis index
IFI	Incremental Fit Index
